# Searching for Noninvasive Predictors of the Diagnosis and Monitoring of Eosinophilic Esophagitis—The Importance of Biomarkers of the Inflammatory Reaction Involving Eosinophils

**DOI:** 10.3390/biom11060890

**Published:** 2021-06-15

**Authors:** Joanna Sarbinowska, Benita Wiatrak, Dorota Waśko-Czopnik

**Affiliations:** 1 Department of Gastroenterology and Hepatology, Wroclaw Medical University, Borowska 213, 50-556 Wroclaw, Poland; sarbinowska.joanna@gmail.com (J.S.); dorota.wasko-czopnik@umed.wroc.pl (D.W.-C.); 2 Department of Pharmacology, Wroclaw Medical University, Mikulicza-Radeckiego 2, 50-345 Wroclaw, Poland

**Keywords:** eosinophilic esophagitis, eotaxin 3, interleukin 5, interleukin 13, major basic protein, transforming growth factor beta 1

## Abstract

Background: Invasive and costly endoscopic diagnosis is obligatory for the diagnosis and monitoring of eosinophilic esophagitis (EoE). This study aims to evaluate the usefulness of serum biomarkers involved in eosinophil-mediated inflammation in the management of EoE. Methods: A prospective cohort study was conducted in 58 patients with dysphagia. Each participant completed a health questionnaire, underwent esophagogastroduodenoscopy with esophageal biopsy for histopathological examination and assessment of total, inflammatory and fibrostenotic Eosinophilic Esophagitis Reference Score (EREFS). Serum levels of interleukin 5 (IL-5), interleukin 13 (IL-13), transforming growth factor β1 (TGF-β1), major basic protein (MBP), and eotaxin 3 were determined by enzyme immunoassays. Total of 16 patients meeting the histological criteria for EoE were treated with proton pump inhibitors for 8 weeks, and then the same diagnostics was performed again. Results: Statistically significantly higher concentrations of MBP and TGF-β1 were demonstrated in the group of patients with EoE, while MBP and eotaxin 3 correlated with the peak eosinophil count (PEC). Baseline MBP levels and eotaxin 3 after treatment significantly positively correlated with EREFS. There was a negative correlation between IL-13 and fibrostenotic EREFS. Additionally, after treatment, a negative correlation TGF-β1 was noted with the inflammatory EREFS and a positive correlation with the fibrostenotic EREFS. Conclusions: The potential role of MBP in predicting the diagnosis of EoE, eotaxin 3 in predicting the advancement and correlation of IL-13 and TGF-β1 in differentiating the inflammatory and fibrotic course of the disease may facilitate the management and individualization of EoE therapy.

## 1. Introduction

In recent decades, in response to global trends aimed at minimizing the invasiveness and the cost of treatments, we observe a significant development of innovative diagnostic methods and technologies to detect new biomarkers based on the study of pathomechanisms of diseases, especially those with a chronic nature and increasing morbidity.

Eosinophilic esophagitis (EoE) is a disease that has undoubtedly been a clinical challenge in the last two decades, not only due to the almost 30-fold increase in the incidence, more than 20-fold increase in the frequency of performing diagnostic tests [1,2], but also due to chronic course and recurrent nature of the disease, often leading to complications [2]. The dynamic development of research on EoE, which was initially considered a purely pediatric disease [3], is reflected in multiple changes to diagnostic and therapeutic guidelines that have been updated six times since the disease’s first reports [4,5,6,7,8,9]. According to the definition published in 2017 and maintained in 2020 in the latest recommendations of the American Gastroenterological Association and the Joint Task Force on Allergy-Immunology Practice Parameters, EoE is the primary immune-mediated esophageal disease manifested by esophageal dysfunction in the form of dysphagia and food impaction [9]. Histologically are observed chronic inflammatory infiltrates with a predominance of intraepithelial eosinophils [9]. Detection of ≥15 eosinophils/HPF (per high power field) in a biopsy of the esophageal mucosa, the coexistence of clinical symptoms and the exclusion of other conditions with systemic or local eosinophilic infiltration are therefore mandatory for the diagnosis of the disease, but also for monitoring the advancement and effectiveness of therapy [7,8,9]. Periodic panendoscopy with the collection of numerous specimens from the esophageal mucosa for histopathological evaluation, although considered the only “gold standard”, is not a perfect solution. Apart from being cost-intensive and invasive, which, together with persistent or recurrent symptoms, significantly deteriorate patients’ quality of life [10], they also carry a high risk of underdiagnosis and therapeutic delay. A too small number of samples taken (less than 6), as well as their incorrect location, limited to only one-half of the esophagus or only lesions (while a normal endoscopic image does not rule out the disease) [7,11,12] significantly reduce the chances of a correct diagnosis. Although precisely specified, the histological criterion is based on the pathologist’s subjective assessment, which, as the research shows, in as many as 22% of cases, may lead to underestimation and erroneous exclusion of the diagnosis [13].

In response to the current needs, numerous studies are being developed to maximize the individualization of EoE management by assessing already recognized methods and completely newly developed and implemented technologies [14]. Among them, a minimally invasive targeting marker would be of strategic importance. Even in the case of non-specific clinical symptoms, it should be able to specifically suggest endoscopic and histopathological diagnostics (which are the only methods that ultimately differentiate from other causes of dysphagia, including neoplasm) and be sensitive enough to replace it in control and monitoring the effectiveness of the therapy [15]. All these criteria would be met by a biochemical marker detectable in the blood of patients. However, no substance with sufficient sensitivity and specificity to be included in the guidelines has yet been identified [7,9].

In this study, an attempt was made to assess the concentrations of serum biomarkers involved in the Th2-dependent immune response, and thus influencing the formation and advancement of EoE. These were the cytokines associated with stimulating intra-tissue migration and degranulation of eosinophils—interleukin 5 (IL-5), interleukin 13 (IL-13), and eotaxin 3, as well as biomarkers involved in increasing muscle reactivity, development of fibrosis, and remodeling—eosinophil major basic protein (MBP) and transforming growth factor β1 (TGF-β1) [16,17,18].

The aim of the study was, therefore, to evaluate the use of serum biomarkers (IL-5, IL-13, eotaxin 3, MBP, and TGF-β1) in the diagnosis and monitoring of EoE by assessing their correlation with the occurrence, as well as endoscopic and histopathological advancement of EoE in patients diagnosed with dysphagia.

This study is not the first attempt to assess the diagnostic and prognostic significance of serum markers with a recognized pathophysiological role in EoE. However, the design of this study was adjusted to consider the main allegations raised in the evaluation of previous studies analyzing the concentration of biomarkers in EoE—the prospective nature of the study was adopted, and the time between taking serum samples and performing endoscopic examinations with biopsies was shortened as much as possible [14].

## 2. Materials and Methods

### 2.1. Study Design and Population

This prospective cohort study was conducted at the Department of Gastroenterology and Hepatology and the Department of Otolaryngology, Head and Neck Surgery at Wroclaw Medical University in Poland. From 1 November 2017 to 30 April 2020, the 58 adult patients were recruited to the project for endoscopic diagnosis of dysphagia. The criterion of exclusion from participation in the study were already diagnosed chronic diseases with possible eosinophilic infiltration of the gastrointestinal tract (eosinophilic esophagitis, eosinophilic gastroenteritis, Crohn’s disease, celiac disease), rheumatological, dermatological, infectious and genetic disorders with possible peripheral eosinophilia, as well as dysphagia caused by a diagnosed neoplastic infiltration of the esophagus. None of the project participants was a transplant recipient, and no one reported heartburn as an accompanying symptom of dysphagia. Before enrollment in the project, high-resolution esophageal manometry (HRM) was performed to rule out patients with achalasia as a potential cause of esophageal eosinophilia. HRM always precedes panendoscopy to avoid the therapeutic effect of the endoscope and possible effects on the manometric parameters and esophageal motility assessment.

Each project participant completed a questionnaire on health and existing diseases, with particular emphasis on atopy. Esophagogastroduodenoscopy was performed, and serum levels of cytokines IL-5 and IL-13, TGF-β1, and eotaxin 3, as well as the product of eosinophil degranulation—MBP, were determined. Diagnostic panendoscopies were performed by one endoscopist—gastroenterology specialist using an Olympus GIF-Q180 device (Olympus, Tokyo, Japan). During the medical examination, the presence of endoscopic features of esophagitis, hiatal hernia, and Schatzki ring were analyzed in detail. Retrospectively, based on the obtained photographic documentation and results description, the presence and advancement of features included in the EoE Endoscopic Reference Score (EREFS) were assessed, including edema, rings, exudates, furrows and strictures, and also crepe paper esophagus, i.e., mucosal fragility or laceration upon passage of diagnostic endoscope [19]. EREFS was assessed similarly to the study by Dellon et al. [20], taking into account the current modified EREFS classification system [19]. Total EREFS (rated on a scale from 0 to 9) was the sum of the points obtained in assessing all the EREFS classification features. The inflammatory subscore was the sum of the points given for the presence of exudate, edema and furrows (from 0 to 4), and fibrostenotic subscore for the diagnosis of rings and strictures (from 0 to 4). Regardless of the presence of the described macroscopic changes, from each participant during the study, six esophageal mucosa biopsy specimens were collected (two each for distal, middle, and proximal esophagus). The obtained material was sent for histopathological examination to assess peak eosinophil count (PEC) at each biopsy, interpreted as the maximum number of eosinophils per HPF (standard size ~0.3 mm^2^). Each biopsy sample was re-verified by a second independent specialist—a pathologist. A venous blood sample was also collected from each participant within a maximum of 7 days after endoscopy, centrifuged, and the collected serum was stored at −70 °C. Quantification of IL-5, IL-13, and TGF-β1 levels was performed using Diaclone enzyme immunoassays (Diaclone SAS, Besancon, France), and eotaxin 3 and MBP using Cloud-Clone enzyme immunoassays (Cloud-Clone Corp., Houston, TX, USA). Both test protocols and reference values of biomarkers for the general population were adopted in accordance with the recommendations of the assay manufacturers (for IL-5 from 0 to 18.49 pg/mL, for IL-13 from 0 to 7.28 pg/mL, for TGF-β1 from 5222 to 13,731 pg/mL, for eotaxin 3 from 18.6 to 51.2 pg/mL, and for MBP from 372.1 to 685.4 ng/mL).

After completing medical examinations, the project participants were divided according to the histopathological criterion’s fulfillment for the diagnosis of EoE. Patients with ≥15 eosinophils/HPF in the biopsy samples constituted the group of patients with EoE, while the remaining patients—the non-EoE group. EoE patients were then treated for 8 weeks with proton pump inhibitor (PPI)—omeprazole in the dose of 20 mg twice daily (following current therapeutic UEG, EAACI ESPGHAN, and EUREOS guidelines from 2017 with later amendments) [7,8,9]. After 8 weeks, each patient in the EoE group completed the health and symptom questionnaire again, had a second panendoscopy with distal, middle, and proximal esophagus biopsies for histopathological examination. Venous blood was collected again to determine eosinophil-mediated inflammatory biomarkers (the protocols were identical to those used for qualifying patients to the project).

### 2.2. Statistical Analysis

The sample size was calculated using the general linear model (α = 0.05; power = 0.90; effect size = 0.25). The required number of patients was calculated as 39. We assumed a dropout rate of 30%, and a sample size of 58 patients was selected.

The data distribution was analyzed with the Shapiro–Wilk test, and it turned out that there was no normal distribution. Data are presented as median and interquartile ranges (IQR). The comparison of demographic data between the group of patients with EoE and the control group was performed using the chi-squared test. Quantitative values obtained in pre-treatment and post-treatment groups of patients were compared using the Wilcoxon test.

One-dimensional logistic models were used to assess the relationships and prediction potential of the studied biomarkers. The dependent variable was the variable representing the diagnosis of EoE, and the independent variable was the biomarker under study. Significant statistical models are marked in red. Sensitivity and specificity were calculated and presented for biomarkers for the EoE pre-diagnosis score. Spearman’s rank correlation coefficients were used to investigate correlations between biomarker levels, PEC in esophageal biopsies and diagnosis of EoE.

Statistical analyses were performed using Statistica 13.0 software (Dell Software Inc., Round Rock, TX, USA). In the data analysis, *p* < 0.05 was used as the level of significance.

### 2.3. Ethical Considerations

The Bioethics Committee at the Wroclaw Medical University approved the project on 17 August 2017 (KB no. 544/2017), with a subsequent extension on 6 December 2018 (KB no. 730/2018). All project participants gave informed written consent to participate in the study.

## 3. Results

### 3.1. Study Population

During the 30 months of the study, taking into account the assumed exclusion criteria, 58 patients were recruited for endoscopic diagnosis due to dysphagia. Based on the first histopathological evaluation of the specimens from the esophageal mucosa collected during esophagogastroduodenoscopy, initially, 15 patients met the histopathological criteria for the diagnosis of EoE. However, in 16 patients (27.6%), EoE features were confirmed by microscopic examination after re-evaluating the specimens. The remaining 42 persons (72.4%) belonged to the non-EoE group, in which 6 patients were diagnosed with hiatal hernia, and 4 persons with erosive esophagitis and Schatzki ring as a possible cause of dysphagia reported upon admission. In 28 participants, the cause of the symptoms was not identified in the endoscopic and histopathological examination.

Despite the disproportion in both groups’ size, no statistically significant differences were observed in terms of age, the burden of atopic diseases or clinical symptoms related to esophageal dysfunction (Table 1). The demographic feature differentiating the studied populations was gender—in the EoE group, a statistically significant majority of patients were men (68.75% vs. 40.48%, *p* = 0.05).

**Figure 1:**
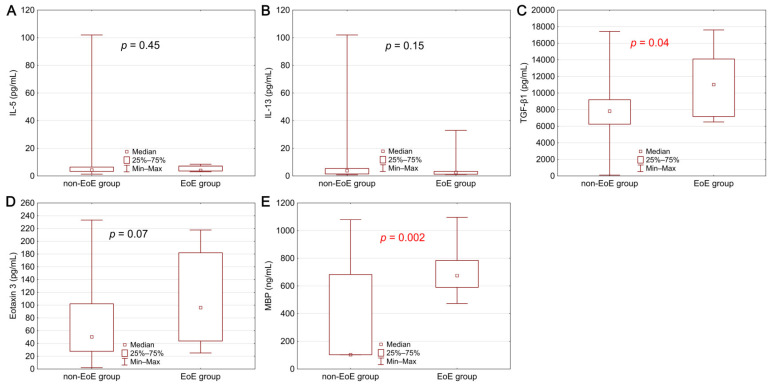
Comparison of serum biomarker concentrations between the group of patients with EoE and the non-EoE group: (**A**) interleukin 5—IL-5, (**B**) interleukin 13—IL-13, (**C**) transforming growth factor β1—TGF-β1, (**D**) eotaxin 3, (**E**) major basic protein—MBP. Statistical significance was evaluated with a Mann–Whitney U test.

The described groups significantly differed in terms of histopathological, endoscopic, and biochemical features (Table 1, Figure 1). As predicted, EoE patients had significantly higher median PEC values than the non-EoE group (*p* = 0.0001). However, endoscopic features of esophagitis (*p* = 0.33), hiatal hernia (*p* = 0.86), and Schatzki’s ring (*p* = 0.12) are not characteristic of patients with dysphagia in the course of EoE in the studied population. Among the six key features included in the EREFS, the presence of edema (*p* = 0.026), as well as all endoscopic features of fibrostenosis, i.e., esophageal rings (*p* = 0.046) and strictures (*p* = 0.02), was significantly more often observed in the group of patients with EoE. The results for both EREFS subscores—inflammatory (*p* = 0.003) and fibrostenotic (*p* = 0.02), and also total EREFS (*p* = 0.0015) turned out to be significantly higher in the EoE group compared to the control group.

### 3.2. Biomarkers in the Prediction of Diagnosis and Histopathological Advancement

The assessment of the concentration of biomarkers of the eosinophil-mediated inflammatory reaction in the blood serum revealed markers that could predict the disease’s diagnosis. The median (IQR) concentrations determined during biomarker diagnostics are as follows: IL-5—4.25 (range 1.30–23.40) pg/mL, IL-13—3.00 (range 0.79–33.00) pg/mL, eotaxin 3—50.85 (range 1.98–233.10) pg/mL, MBP—682.5 (range 299.0–1096.0) ng/mL and TGF-β1—7995 (range 3150–17,604) pg/mL.

The concentration of the studied biomarkers was compared between the group of patients with EoE and the control group. Statistically significantly higher concentrations of MBP (*p* = 0.002) and TGF-β1 (*p* = 0.04) were demonstrated in the EoE patients (Figure 1). A higher level was also observed in the case of eotaxin 3, where the difference was close to statistical significance (*p* = 0.07).

Similar results were obtained from the analysis in terms of exceeding the reference values of individual biomarkers. Obtained serum levels of TGF-β1 (*p* = 0.04) and MBP (*p* = 0.0001) exceed the upper limit of the general population’s reference values. The described dependence was also observed for eotaxin 3, but without statistical significance (*p* = 0.31).

Relationships between biomarkers, diagnosis of EoE and PEC were evaluated using Spearman’s rank correlation coefficients (Table 2). Levels of IL-5 and IL-13 showed a positive, statistically significant correlation between them. Simultaneously, there were weak negative correlations between these cytokines and PEC, diagnosis of EoE and concentrations of other biomarkers (for eotaxin 3 and in the case of IL-13 vs. TGF-β1 statistically significant). The concentration of TGF-β1 significantly correlated with the diagnosis of EoE and showed a weak positive correlation with the PEC. The opposite situation was observed for eotaxin 3, which significantly correlated with the PEC, without a significant positive correlation with the EoE diagnosis. The strongest statistically significant correlation was obtained for MBP, both with the PEC and diagnosis, which indicates the potential importance of this biomarker in diagnosing EoE.

In addition to assessing the possible role of biomarkers in predicting the diagnosis of EoE, this study also attempts to assess their importance in predicting histological remission. For this purpose, the concentrations of biomarkers obtained from patients with EoE after 8 weeks of PPIs therapy were correlated with the PEC in samples collected from the esophageal mucosa during the control esophagogastroduodenoscopy. Due to the invasiveness and nuisance of the follow-up examination and limited endoscopic control during the COVID-19 pandemic, only 7 patients (i.e., 43.75% of project participants diagnosed with EoE) participated in the re-evaluation after two months of treatment. Among them, 5 patients (71.43%) achieved histopathological remission, defined as a reduction in the number of eosinophils found in esophageal mucosa biopsies below 15/HPF (median 10, range 0–70 eosinophils/HPF). The median (IQR) concentrations of the biomarkers after treatment are as follows: IL-5—5.8 (range 3.2–8.8) pg/mL, IL-13—4.1 (range 1.4–26.2) pg/mL, eotaxin 3—61.3 (range 34.9–120.7) pg/mL, MBP—577 (range 349–637) ng/mL, and TGF-β1—6690 (range 5670–15,024) pg/mL. Analysis of these values showed a strong positive but not statistically significant correlation of TGF-β1, eotaxin 3, and MBP with PEC value (Table 2). The correlation of these markers with diagnosis and histopathological advancement was thus confirmed both at the diagnosis of EoE and after the first 8 weeks of treatment, but statistically significant values were obtained only in the first examination (Table 2).

### 3.3. Biomarkers in the Assessment of Endoscopic Advancement and Prognosis of Inflammatory or Fibrostenotic Course

In addition to the possible diagnostic potential in predicting histopathological advancement of EoE, the usefulness of the studied biomarkers in correlation with an endoscopic assessment of total, inflammatory and fibrostenotic EREFS was also checked (Table 3).

MBP was a marker most strongly (statistically significantly) correlated with eosinophilic infiltration and endoscopic advancement in all EREFS subscores. Contrary to the results obtained before treatment, the correlation with fibrostenotic EREFS after treatment was weak negative (but statistically significant). IL-13 significantly correlated only with post-treatment fibrostenotic EREFS, and this correlation was strong negative. The moderate negative correlation between IL-13 and total EREFS after treatment is also noteworthy. A relationship pattern opposite to MBP after treatment was observed for TGF-β1 after treatment—there were significant weak correlations, negative with inflammatory EREFS and positive with fibrostenotic EREFS. After treatment, positive statistically significant correlations were obtained for eotaxin 3—moderate for inflammatory EREFS, weak in the fibrostenosis, and strong for a total score. In the case of IL-5, only weak and statistically insignificant correlations with EREFS were observed, which does not allow including this interleukin among the markers of prognostic importance in assessing endoscopic advancement.

### 3.4. Diagnostic Potential of the Studied Biomarkers

**Figure 2:**
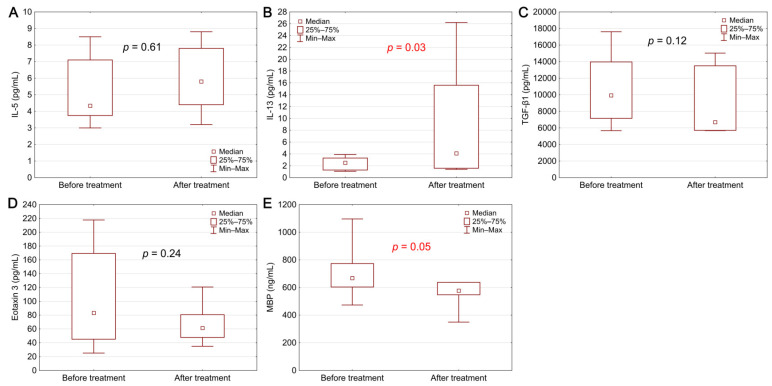
Biomarker levels in EoE patients before and after treatment: (**A**) IL-5, (**B**) IL-13, (**C**) TGF-β1, (**D**) eotaxin 3, (**E**) MBP. Statistical significance was evaluated with a Wilcoxon test.

Biomarker concentrations before diagnosis and after 8 weeks of therapy in the group of patients with EoE are presented in Figure 2. After treatment with PPI, a statistically significant decrease in MBP concentration was observed (*p* = 0.05). The treatment also caused an increase in the IL-13 level (*p* = 0.03).

**Figure 3:**
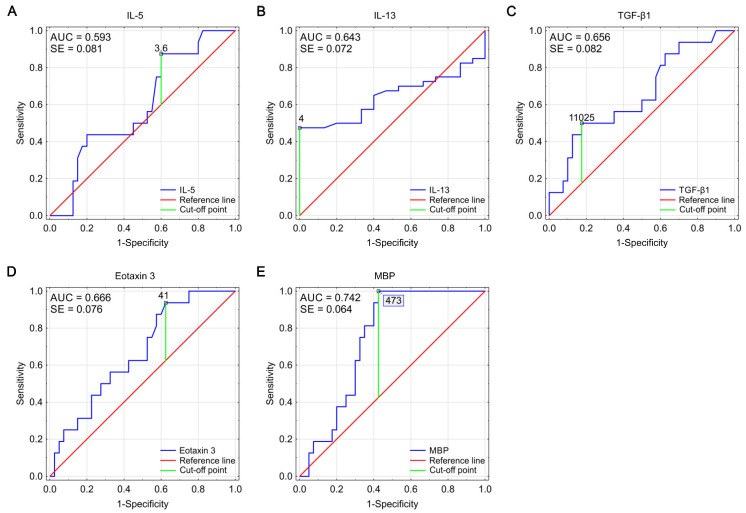
ROC curves for IL-5 (**A**), IL-13 (**B**), TGF-β1 (**C**), eotaxin 3 (**D**), and MBP (**E**), showing their effectiveness as markers predicting the diagnosis of EoE. Optimal cut-off points were determined using Youden index analysis (AUC—area under the curve, SE—standard error).

A graphical representation of the effectiveness of studied biomarkers in predicting EoE diagnosis is presented in Figure 3 as ROC curves. The calculated AUC values (area under the ROC curve) for all markers oscillated in the range of 0.593–0.742. The highest AUC value was obtained for the MPB, simultaneously with the lowest AUC error value.

## 4. Discussion

So far, many studies have attempted to identify a tissue marker correlating with diagnosis [21], progression [22,23,24,25], and response to EoE treatment [22,26,27], allowing for the differentiation of esophageal diseases with accompanying dysphagia [28,29,30] and being a trigger marker in disease development, and thus an effective target of biological therapies [31]. Due to the predicted low specificity of markers involved simultaneously in the pathomechanisms of numerous allergic diseases [32] and the ambiguous results of research on tissue markers in EoE, little attention was paid to assessing the significance of these markers’ serum levels.

In this study, we looked for a minimally invasive marker, determined in venous blood serum, having a potential predictive value for the diagnosis, histopathological and endoscopic advancement of EoE, and correlated with the response to PPI treatment.

Based on the results of our study, it can be concluded that MBP was a serum marker most strongly (statistically significantly) correlated with both the diagnosis of EoE, as well as the peak number of eosinophils/HPF and endoscopic advancement (assessed at diagnosis by inflammatory, fibrostenotic, and total EREFS). The highest sensitivity and specificity also characterized this marker. Although the correlation between the level of MBP in blood serum [33] or saliva of patients [34] and the diagnosis or stage of EoE has not been proven so far, this marker’s importance in the esophageal string test has been repeatedly emphasized [35,36], and above all in tissue tests. Positive correlations were found in predicting the diagnosis of EoE [28,29] and in assessing the response to treatment [26]. The advantage of MBP1 over the peak eosinophil count (PEC) in diagnosing the disease was also proven in two research studies based on the assessment of tissue markers [23,37]. This was justified pathophysiologically by the degranulation of eosinophils, which by releasing granular proteins, including MBP, into the tissues, lose their cellular morphological phenotype and therefore are not included in the histopathological examination result [23,37]. The correlation between MBP and the diagnosis of EoE found in our study, although strong and statistically significant, is weaker than the correlation between the diagnosis and PEC. This is probably the price of less invasive serological determinations, but in the face of a limited number of studies on this group of potential predictors of EoE diagnosis, it does not undermine the sense of the study.

A serum marker that was also strongly correlated with PEC in our study was eotaxin 3, while the level of TGF-β1 correlated with the diagnosis of EoE. For these three markers, i.e., MBP, eotaxin 3, and TGF-β1, there were also strong positive, but not statistically significant, correlations with the PEC after 8-week PPI therapy. Due to the small group of patients with EoE recruited to the project, low attendance (43.75%) in control studies after 8-week PPI therapy, as well as the lack of statistical significance of the correlations between markers and PEC after treatment, it is difficult in this study to select a serum marker predicting the histological remission of the disease.

The levels of MBP, TGF-β1, and eotaxin 3 were positively correlated with each other. In turn, a negative correlation occurred between these markers and IL-5 and IL-13 cytokines (with a positive correlation between them). It can be interpreted as a synergy of these proteins’ actions at subsequent stages of developing the inflammatory reaction involving eosinophils. The pathophysiology of the disease confirms this. After the significant participation of IL-5 and IL 13 in the stimulation of the influx of eosinophils to the esophageal mucosa, with the development of inflammation, their importance and tissue concentration decrease, and secondarily also their concentration in the blood serum. They give way to induced eosinophil-activating chemokines, such as eotaxin 3, and products of eosinophil degranulation, including MBP and TGF-β [38].

Considering the small number of studies on blood serum markers to date, an attempt to predict the course and advancement of inflammatory and fibrostenotic EoE based on the correlation with the endoscopic assessment of EREFS seems innovative. Apart from the already discussed correlation with MBP, we also observed strong and moderate statistically significant correlations of eotaxin 3 with remission scores in each of the post-treatment EREFS subscores in our study. The inhibitory effect of treatment with conventional doses of PPIs on the expression of eotaxin 3, and secondarily on the development of the disease [39,40,41], would therefore be reflected in the results of this study and would settle the hitherto ambiguous observations confirming [42] or denying [33,43,44] the importance of eotaxin 3 concentration in monitoring the course of EoE.

Based on the interpretation of the IL-13 and TGF-β1 concentrations, it seems possible to differentiate the course of the inflammatory and fibrostenotic EoE in the studied group of patients. The increase in the concentration of TGF-β1, with the simultaneous decrease in the concentration of IL-13 in the serum, may correspond to the development of fibrostenosis in the course of EoE. Conversely, a low concentration of TGF-β1 in the serum, with a simultaneous increase in the concentration of IL-13, may indicate less advanced disease and the predominance of inflammatory processes over fibrostenotic processes. These correlations were observed despite the apparent individual low specificity of both TGF-β1 and IL-13 in the diagnosis and monitoring of EoE [33]. TGF-β1 is considered the “main mediator of fibrosis” responsible for the activation of fibroblasts and the induction of epithelial-mesenchymal transformation in many fibrostenotic processes [45]. In turn, IL-13 is well-known for its role in many atopic diseases, where it contributes to eosinophil chemotaxis, goblet cell hyperplasia, collagen deposition and an increase in smooth muscle contractility [17].

Another investigated serum marker with a confirmed role in the pathomechanism of EoE is IL-5. Previous studies assessing the importance of this cytokine in diagnosing and monitoring EoE have not confirmed the correlation of its concentration with the diagnosis and course of the disease in the group of adult patients [34] and the pediatric population [46]. In another prospective study evaluating serum biomarker levels in EoE after PPIs therapy, a statistically significant negative correlation was found between IL-5 and esophageal eosinophilia and no prediction of the post-treatment tissue eosinophilia [44]. Similar conclusions can be drawn from this study, but the negative correlation with esophageal eosinophilia was not statistically significant. In the cited study, the described relationship was justified by the high accuracy of the ELISA test used [44], which may also be reflected in our study (at the detection threshold of 5 pg/mL, the median IL-5 concentration in the group of patients with EoE was 4.07 pg/mL and 4.30 pg/mL in the control group). A negative correlation with tissue eosinophilia can also be observed in the case of IL-13, the concentration of which, similarly to IL-5, significantly increases in the serum after treatment. The described observation is not entirely clear but may be due to the mediation of these interleukins in the remodeling process, leading to esophageal motility disorders, which may persist regardless of the active eosinophilic inflammation, even after its complete resolution [47,48,49].

This study’s undoubted advantage is an attempt to minimize the invasiveness of diagnosis and monitoring of EoE by evaluating the so far rarely assessed or not assessed markers in blood serum with a confirmed pathophysiological relationship with EoE. Important aspects are also: the prospective nature of the study, the shortest possible time interval between taking serum samples and performing endoscopic examinations with biopsies, re-verification of all histopathological examinations of the specimens collected during the project, as well as the correct selection of the study population—homogeneous in terms of age and symptoms, allergic burden, and heterogeneous only in terms of gender. Male gender is a significant risk factor for EoE resulting from the suggested sex-dependent association between single nucleotide polymorphisms in the thymic stromal lymphopoietin gene and its receptor and the protective effect of estrogen hormone signaling in women [7]. The weakness of this study is the relatively small study group, the population limited to adults only, and the lack of a pH-metric assessment that would allow for objective classification of patients with possible gastroesophageal reflux, often coexisting with EoE or being an independent cause of dysphagia in the group of patients without EoE diagnosis. The limitations of this project suggest the need to continue research on noninvasive blood serum biomarkers and confirm the obtained results in the validation cohort, taking into account the possible effect of co-occurrence and overlapping of EoE and GERD, as well as depending on the pharmacotherapy used: PPI, local steroid therapy or elimination diet.

## 5. Conclusions

Based on the results of this study and the available literature data, it is not possible to select one serum biomarker with pleiotropic predictive and prognostic functions in EoE.

The observed trend, suggesting the importance of MBP in predicting the diagnosis and eotaxin 3 in predicting disease advancement, emphasizes the potential for improving the management and increasing the individualization of treatment. However, the necessary condition is to determine the markers several times, and not one parameter should be considered, but the whole group of them together, taking into account the pathophysiological role and interdependencies.

It can be predicted that this project, as well as the existing high-quality prospective studies correlating the concentration of individual markers in the blood serum with the diagnosis and progression of EoE, has developed a material for the creation of an automated algorithm that would provide intelligent analysis of the obtained data and could improve the precision of EoE diagnostics and therapy in the future.

## Figures and Tables

**Table 1:** Characteristics of the study participants divided into the group with eosinophilic esophagitis (EoE) and without EoE (EREFS—Eosinophilic Esophagitis Endoscopic Reference Score, PEC—peak eosinophil count).

Parameters		EoE	Without EoE	*p*
Patients [*n* (%)]		16 (27.6)	42 (72.4)	-
Age median (range)		28.5 (20–50)	36.5 (24–68)	0.47
Male [*n* (%)]		11 (68.75)	17 (40.48)	0.05
Atopy [*n* (%)]		8 (50.00)	20 (47.62)	0.87
Atopy	inhalation allergies [*n* (%)]	4 (25.00)	10 (23.81)	0.92
	food allergies [*n* (%)]	4 (25.00)	5 (11.90)	0.21
	bronchial asthma [*n* (%)]	0 (0.00)	5 (11.90)	0.14
	atopic dermatitis [*n* (%)]	2 (12.50)	3 (7.14)	0.52
	allergic sinusitis [*n* (%)]	1 (6.25)	3 (7.14)	0.90
Clinical symptoms	choking [*n* (%)]	9 (56.25)	18 (42.86)	0.36
	food impaction [*n* (%)]	9 (56.25)	21 (50.00)	0.67
	odynophagia [*n* (%)]	7 (43.75)	18 (42.86)	0.95
Endoscopic features	inflammation [*n* (%)]	3 (18.75)	4 (9.52)	0.33
	endoscopic features of a hiatal hernia [*n* (%)]	2 (12.50)	6 (14.29)	0.86
	Schatzki ring [*n* (%)]	4 (25.00)	4 (9.52)	0.12
	edema [*n* (%)]	6 (37.50)	5 (11.90)	0.026
	rings [*n* (%)]	8 (50.00)	10 (23.38)	0.046
	exudates [*n* (%)]	3 (18.75)	5 (11.90)	0.49
	furrows [*n* (%)]	3 (18.75)	3 (7.14)	0.19
	strictures [*n* (%)]	1 (12.50)	0	0.02
	crepe paper esophagus [*n* (%)]	0	0	-
EREFS	inflammatory [*n* (%)]	9 (56.25)	8 (19.04)	0.003
	fibrostenotic [*n* (%)]	9 (56.25)	10 (23.81)	0.02
	total [*n* (%)]	12 (75.00)	12 (28.57)	0.0015
PEC	median (range)	45 (15–100)	0 (0–5)	0.0001

**Table 2:** Spearman’s rank correlation coefficients between biomarkers, diagnosis and PEC, before and after treatment.

Parameters	Before Treatment (*n* = 16)						After Treatment (*n* = 7) PEC
	PEC	Diagnosis of EoE	IL-13	IL-5	TGF- β1	Eotaxin 3	
IL-13	−0.12	−0.19					0.02
IL-5	−0.04	0.10	0.42				0.71
TGF-β1	0.10	0.27	−0.33	−0.12			0.53
Eotaxin 3	0.33	0.24	−0.46	−0.15	0.08		0.66
MBP	0.53	0.41	0.03	−0.13	0.06	−0.01	0.43

**Table 3:** Spearman’s rank correlation coefficients between biomarkers and EREFS subscores, before and after treatment.

	Parameter	IL-13	IL-5	TGF-β1	Eotaxin 3	MBP
Before Treatment (*n* = 16)	Inflammatory EREFS	0.021	−0.037	0.145	−0.014	0.526
	Fibrostenotic EREFS	−0.134	−0.142	0.226	0.106	0.264
	EFERS	−0.063	−0.084	0.224	0.094	0.447
After Treatment (*n* = 7)	Inflammatory EREFS	0.144	0.000	−0.144	0.577	0.874
	Fibrostenotic EREFS	−0.722	0.289	0.289	0.289	−0.291
	EFERS	−0.535	0.267	0.134	0.802	0.539

## Data Availability

The data generated and analyzed during the current study are available from the corresponding author upon reasonable request.

## Trial Registration

This trial was registered with ClinicalTrials.gov (no. NCT04803162). Registered 17 March 2021—Retrospectively registered, https://clinicaltrials.gov/ct2/show/NCT04803162 (accessed on 13 June 2021).

## Abbreviations

AUC	area under the curve
ELISA	enzyme-linked immunosorbent assay
EoE	eosinophilic esophagitis
EREFS	Eosinophilic Esophagitis Endoscopic Reference Score
HPF	high power field
IL-5	interleukin 5
IL-13	interleukin 13
IQR	interquartile ranges
MBP	major basic protein
PEC	peak eosinophil count
PPI	proton pump inhibitor
ROC	receiver operating characteristic
TGF-β1	transforming growth factor β1
